# Consortium of Plant Growth-Promoting Rhizobacteria Strains Suppresses Sweet Pepper Disease by Altering the Rhizosphere Microbiota

**DOI:** 10.3389/fmicb.2019.01668

**Published:** 2019-07-23

**Authors:** Li-Na Zhang, Da-Cheng Wang, Qiang Hu, Xiang-Qun Dai, Yue-Sheng Xie, Qing Li, Hua-Mei Liu, Jian-Hua Guo

**Affiliations:** ^1^Department of Plant Pathology, College of Plant Protection, Nanjing Agricultural University, Key Laboratory of Monitoring and Management of Crop Diseases and Pest Insects, Ministry of Agriculture, Engineering Center of Bioresource Pesticide in Jiangsu Province, Nanjing, China; ^2^Wuhan Kernel Bio-tech Co., Ltd., Wuhan, China

**Keywords:** BBS, rhizosphere soils, sweet pepper, disease prevalence, soil properties

## Abstract

Beneficial microorganisms have been extensively used to make plants more resistant to abiotic and biotic stress. We previously identified a consortium of three plant growth-promoting rhizobacteria (PGPR) strains (*Bacillus cereus* AR156, *Bacillus subtilis* SM21, and *Serratia* sp. XY21; hereafter “BBS”) as a promising and environmentally friendly biocontrol agent. In this study, the effect of BBS on a soil-borne disease of sweet pepper was evaluated. Application of BBS significantly reduced the prevalence of phytophthora blight and improved fruit quality and soil properties relative to the control. BBS was able to alter the soil bacterial community: it significantly increased the abundances of *Burkholderia*, *Comamonas*, and *Ramlibacter*, which were negatively associated with disease severity, relative to the control. A redundancy analysis suggested that BBS-treated soil samples were dominated by *Burkholderia*, *Comamonas*, *Ramlibacter*, *Sporichthya*, *Achromobacter*, and *Pontibacter*; abundance of these genera was related to total organic carbon (TOC), total nitrogen (TN), ammonium nitrogen (AN), total potassium (TP), and available phosphorus (AP) contents. This suggests that BBS treatment shifted the microbe community to one that suppressed soil-borne disease and improved the soil chemical properties.

## Introduction

Sweet pepper *Capsicum annuum* L. var. grossum (Solanaceae) is an annual plant cultivated throughout the world. It is widely valued because of its unique flavor and high nutritional value, especially in terms of its vitamin C content. With the expansion of modern facilities and high-efficiency factory farms, sweet pepper cultivation has increased dramatically where cultivated land has expanded (Shu et al., 2016). Global production of chilies and peppers was at 34.5 million tons from 1.9 million ha of crop-growing surface area in 2016; China was the largest contributor, producing 17.45 million tons from 0.75 million ha of land (FAO, 2018).

However, soil-borne diseases such as phytophthora blight have increased through the practice of continuous cropping (Li et al., 2017). Meanwhile, these diseases cause the loss of soil quality which have severely restricted the development of the sweet pepper industry (Zhang et al., 2016) and the income of sweet pepper growers has also been reduced. Farmers have therefore increased the frequency of pesticide application to enhance yields. However, massive application of chemical pesticides has caused many negative impacts, such as the contamination of food, soil, and water by pesticide residues, and loss of biodiversity. Besides, excessive use of chemical pesticides destabilizes the soil micro-ecosystem, and this is an important cause of soil-borne diseases (Avis et al., 2008). The majority of soil-borne pathogens survive in bulk soil; under suitable conditions, they infect host plants to establish parasitic relationships with the plants (Raaijmakers et al., 2009; Liu et al., 2018).

Previous studies have reported that beneficial microbes can be recruited by host plants to counteract pathogen infection (Dudenhöffer et al., 2016). For instance, beneficial microbiomes can induce disease resistance in plants to many plant pathogens such as *Ralstonia solanacearum* (Aliye et al., 2008; Cao et al., 2018), *Phytophthora capsici* (Lim and Kim, 2010; Sang et al., 2018), and *Botrytis cinerea* (Nie et al., 2017; Jiang et al., 2018). Beneficial microbiomes improve soil chemical properties and fruit quality (Song et al., 2015). Furthermore, soil inoculation by microbes alters the resident microbial communities (Trabelsi and Mhamdi, 2013). Zhang et al. (2019) reported that inoculation with *Bacillus velezensis* NJAU-Z9 to pepper led to a higher rhizosphere bacterial richness and diversity compared to the control without NJAU-Z9 inoculation. In addition, recent work has shown that managing rhizosphere microbial communities contributes to plant disease control (Mazzola, 2010). Wang et al. (2015) revealed that inoculation using bio-organic fertilizer reduced the prevalence of tomato fusarium wilting by altering the soil microbial communities. Liu et al. (2018) found that bio-organic additives (matured chicken manure added with amino acids and PGPR strain *Bacillus amyloliquefaciens* SQR9) suppressed tomato disease by altering bacterial community composition in the rhizosphere. Xue et al. (2015) reported that *B. amyloliquefaciens* NJN-6, combined with compost promoted alteration of the rhizosphere bacterial community structure by developing beneficial strains that dominated the microbial community, which contribute to Panama disease control.

Our objective was to evaluate the suppression of sweet pepper disease using a microbial additive (“BBS”) that we have previously developed (Wang et al., 2012; Yang et al., 2014). However, the mechanisms by which soil inoculation by microbes alters rhizosphere microflora to control sweet pepper diseases are still not well understood. Therefore, we conducted a 3-year field experiment (2014–2016) in sweet pepper producing areas in China where soil-borne diseases were prevalent year-round. Specifically, the soil-borne disease pepper blight caused by *Phytophthora capsici* has posed a serious threat to sweet pepper production in these areas. Using sequencing, we then surveyed the rhizosphere microbiota, to assess the effects of the microbial additive and to examine the relationships between the rhizosphere microbiota and the plant disease.

## Materials and Methods

### Bacterial Strains and Culture Conditions

Three PGPR strains (*Bacillus subtilis* SM21, *Bacillus cereus* AR156, and *Serratia* sp. XY21) were cultured at 28°C for 24 h on Luria-Bertani (LB) agar medium. A single colony from a freshly streaked plate was then selected, inoculated into LB broth, and incubated at 28°C for 48 h in a shaker at 200 rpm. The broth culture was spun at 6000 × *g* in a centrifuge for 15 min, and the resulting pellet was resuspended in sterile water and adjusted to a concentration of 10^9^ colony forming units per ml (CFU/ml) for further experiments.

### Field Experimental Design

The field experimental site was located in Huaian, Jiangsu Province, China (33°35′ < 42″N, 119°02′11″E), which has a subtropical monsoon climate with an average annual temperature and precipitation 14.2°C and 940 mm, respectively. The field was continuously utilized for sweet pepper cultivation for several years before our study. Soil-borne diseases pepper blight caused by *P. capsici* has posed a serious threat to sweet pepper production. The soil was a sandy loam with pH 7.08, 80.76 g/kg total organic carbon (TOC), 139.23 g/kg total organic matter (TOM), 12.51 g/kg total N (TN), 26.24 mg/kg NH4^+^-N (AN), 435.10 mg/kg NO3^–^-N (NN), 4.95 g/kg total P (TP), 1.15 g/kg available P (AP), and 1.69 g/kg available K. The field experiment was carried out from December 2013 to April 2016. We define “season” as the entire sweet pepper growing season (from early December to mid-April of next year). Plants were cultivated with or without BBS treatment. Each treatment had three randomized independent replications with a single plot of 6 m × 8 m in area. We applied 500 ml of BBS suspension (1:100 dilution) to each seedling at transplanting; the control seedlings were mock inoculated with an equal volume of water. A five-point (each point is 1.2 m × 1.2 m) sampling method was used for random sampling.

### Assay of Disease Prevalence and Yield

The prevalence of phytophthora blight was investigated 60 days after transplanting into the field. Five points sampling method was used for random sampling. Sixteen pepper samples were collected at each point 1.2 m × 1.2 m, the diseased plants were counted, and prevalence was calculated as follows:

Prevalence=∑numberofdiseaseplants/ total⁢plants⁢investigate×100%

To measure total sweet pepper yield, all mature sweet peppers were harvested and weighed.

### Assay of Leaf Chlorophyll Content

Leaf chlorophyll content was measured 60 days after transplanting using a modified version of the method of Jiang et al. (2014). Chlorophyll extraction was conducted using 80% acetone solution (v/v in water); from each sample, 10.0 g plant tissue was cut into 0.5 cm segments and homogenized with acetone solution at −10°C. The mixture was centrifuged at 12,000 × *g* for 15 min, and the supernatant was transferred to the flask and covered with aluminum. Absorption of the supernatant was measured at 645 and 663 nm using a HITACHI U-2000 spectrophotometer.

### Assay of Fruit Quality

To evaluate sweet pepper quality, the contents of soluble sugar, soluble solids, and vitamin C were assayed at the harvest time. Soluble sugar was determined according to Horowitz and Reynolds (1936). Briefly, 1.0 g fresh fruit was kept in 10 ml of 90% ethanol for 1 h at 60°C in an incubator. The extract was then transferred into a new flask and the final volume was made up to 25 ml by adding 90% ethanol. 1 ml aliquot was transferred to a test tube and 1.0 ml of 5% phenol was added to it and mixed thoroughly, 5 ml of analytical grade sulphuric acid was then added to it and mixed thoroughly by vertical agitation with a glass rod. For exothermic reaction the test tube was cooled in the air. Absorbance was recorded at 485 nm. Soluble solids were measured using a handheld refractometer at 20°C (Barrett et al., 1998). Vitamin C content was detected according to Santos et al. (2016), 2% oxalic acid was used for extraction and the 2,6-dichlorophe-nolindophenol dyestuff was added for reduction. Xylene was used for extracting of the excess dyestuff. Absorbance was recorded at 500 nm.

### Assay of Soil Properties

Soil total organic carbon (TOC) and total organic matter (TOM) were measured by potassium dichromate (K_2_Cr_2_O_7_) oxidation-reduction titration (Schollenberger, 1931). The content of total nitrogen (TN) was determined using the Kjeldahl method (Page et al., 1982). Soil nitrate nitrogen (NN), ammonium nitrogen (AN) and total potassium (TP) content was quantified using an AutoAnalyzer 3 (Bran and Luebbe GmbH, Germany). To determine soil available phosphorus (AP), we followed the molybdenum-blue method using sodium bicarbonate (Olsen, 1954). Soil total potassium (TK) content was detected by atomic absorption spectrophotometry (ASS), and soil available potassium (AK) was measured in the extract using a flame atomic absorption spectrophotometer (Brown, 1998).

### Soil Sampling, DNA Extraction

Both diseased and healthy plants with tightly adherent rhizosphere soil were sampled (Morris et al., 1997). In brief, for each replicate, a five-point sampling method was applied (each point is 1.2 m × 1.2 m). For each point, 16 plants were randomly selected. Eighty plants from five points were collected. After careful removal of 0–5 cm of the topsoil, rhizospheres were excavated, with as much of their associated roots as possible, by digging to a 5–20 cm depth around pepper plants. Soils and Plants were placed into plastic bags and placed on ice for transport to the laboratory for preparations using standard procedures within a few hours. Subsequently, excess bulk soil was removed from the roots by shaking, brushing down firmly adhering soil with sterile brushes, which was defined as rhizosphere soil (Mendes et al., 2017). All of these soils were divided into subsamples: one was frozen at −80°C for DNA extraction and subsequent molecular analysis, the rest was further air-dried at room temperature and passed through a 0.25 mm sieve for chemical analysis. DNA extraction was performed using FastDNA^®^ SPIN Kit (MP Biomedicals, Solon, OH, United States) following the manufacturer’s instructions. The concentration and quality of the DNA samples were evaluated using a NanoDrop 1000, Spectrophotometer (United States). We ensure that adequate amounts of high-quality genomic DNA had been extracted (>90 μg/μl) and no DNA was detected in the negative controls.

### MiSeq Illumina Sequencing

The DNA extracted from each soil sample served as the template for 16S rRNA gene fragment amplification. V3-V4 regions of the bacterial 16S rRNA gene were amplified using primers (PAGE purified) 338F (5′-ACTCCTACGGGAGGCAGCA-3′) and 806R (5′-GGACTACHVGGGTWTCTAAT-3′) (Dennis et al., 2013). The 16S rRNA V3–V4 amplicon was amplified using KAPA HiFi Hot Start Ready Mix (2×) (TaKaRa Bio Inc., Japan). Reaction was set up as follows: microbial DNA (5 ng/μl) 2 μl; amplicon PCR forward primer (1 μm) 5 μl; amplicon PCR reverse primer (1 μm) 5 μl; 2 × KAPA HiFi Hot Start Ready Mix 13 μl (total 25 μl). The plate was sealed and PCR performed in a thermal instrument (Applied Biosystems 9700, United States) using the following program: one cycle of denaturing at 95°C for 3 min, followed by 25 cycles of denaturing at 95°C for 30 s, annealing at 55°C for 30 s, elongation at 72°C for 30 s, and a final extension at 72°C for 5 min. Each sample had three replicates. PCR products were examined on a 2% (w/v) agarose gel, and the band was extracted and purified with the AxyPrep DNA Gel Extraction Kit (Axygen Biosciences, Union City, CA, United States) according to the manufacturer’s instructions and quantified using QuantiFluor^TM^-ST (Promega, United States). Purified amplicons were pooled in equimolar and paired end sequenced (2 × 300) on an Illumina MiSeq platform according to the standard protocols. The programs of amplification and sequencing were carried on using the Illumina MiSeq platform (United States) at BGI Co., Ltd. (Shenzhen, China). All read sequences were deposited in the Sequence Read Archive (SRA) NCBI database (accession number PRJNA526286).

### Bioinformatics Analysis

Raw sequence processing, quality control, and annotation were carried out according to Huang et al. (2015). The representative sequences of bacterial 16S rRNA gene were assigned to taxonomic classifications from genus to phylum at hierarchical levels by RDP Classifier (v2.2) against the Greengene (v201305) with a confidence threshold of 80%. The obtained bacterial OTUs were further modified and only OTUs with >20 counts summed across all samples were retained. The number of sequences per sample ranged from 54 235 to 54 784 (Supplementary Table S1). These were resampled to a depth of 54 235 sequences using the program MOTHUR (v1.31.2), and the resulting new operational taxonomic unit (OTU) table was used for further analyses. Rarefaction analysis was performed by MOTHUR (v1.31.2), and the Observed species, Chao1 and ACE richness estimations, Coverage and the Shannon and Simpson diversities were calculated by MOTHUR (v1.31.2) (Schloss et al., 2009). Principal co-ordinate analysis (PCoA) based on the Bray–Curtis distance metric was carried out using mothur to compare the bacterial microbial community structure among the soil samples (Liu et al., 2016). Spearman’s rank correlation coefficient was used to assess the correlations between selected rhizosphere soil genera abundance and *P. capsici* prevalence. Differentially abundant taxa and OTUs between two microhabitats were calculated using moderated *t*-tests. The resulting *p*-Values were adjusted for multiple hypotheses testing using the Benjamini-Hochberg correction. Heatmap figures were implemented by R (v3.4.2) packages heatmap. The Mantel test was conducted to reveal the relationships between the selected soil properties and rhizosphere soil microbial genera. In addition, redundancy analysis (RDA) was performed to evaluate the relationships among the soil samples, soil properties and rhizosphere soil microbial genera. In addition, Principal co-ordinates analysis (PCoA) and redundancy analysis (RDA) diagrams were generated using R (v3.4.2) package vegan to demonstrate the clustering of different samples.

### Statistical Analysis

Differences between treatment groups were determined statistically at the factorial level by analysis of variance (ANOVA). Differences were considered significant at *P* < 0.05 or *P* < 0.01, and differences among treatments were analyzed via Tukey’s Studentized Range (HSD) test.

## Results

### Effect of BBS on Disease Control and Yield

**FIGURE 1:**
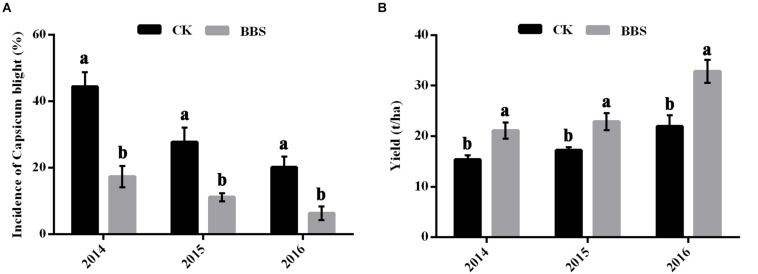
Effects of different fertilization management programs on disease **(A)** and yield **(B)** of sweet pepper in 2014–2016. Data are expressed as the mean ± SD (*n* = 3). Significant differences between different treatments are indicated as different letters on top of the data bars. The statistical analysis was determined by a Tukey’s Studentized Range (HSD) test: α = 0.05, *n* = 3.

The prevalence of phytophthora blight among BBS-treated plants was significantly lower than among the control plants in all three seasons, at 17.36% (2014), 11.11% (2015), and 6.25% (2016) in BBS-treated plants, versus 44.44% (2014), 27.78% (2015), and 20.14% (2016) in the control (Figure 1A). In contrast to the diseased control plants, sweet pepper yields under BBS treatment were significantly higher in all seasons, at 21.10% (2014), 22.88% (2015), and 32.87% (2016) in BBS-treated plants, versus 15.35% (2014), 17.26% (2015), and 21.93% (2016) in the control (Figure 1B). Therefore, BBS treatment progressively reduced the prevalence of soil-borne disease and increased sweet pepper crop yields relative to the control.

### Effect of BBS on Chlorophyll and Fruit Quality

**FIGURE 2:**
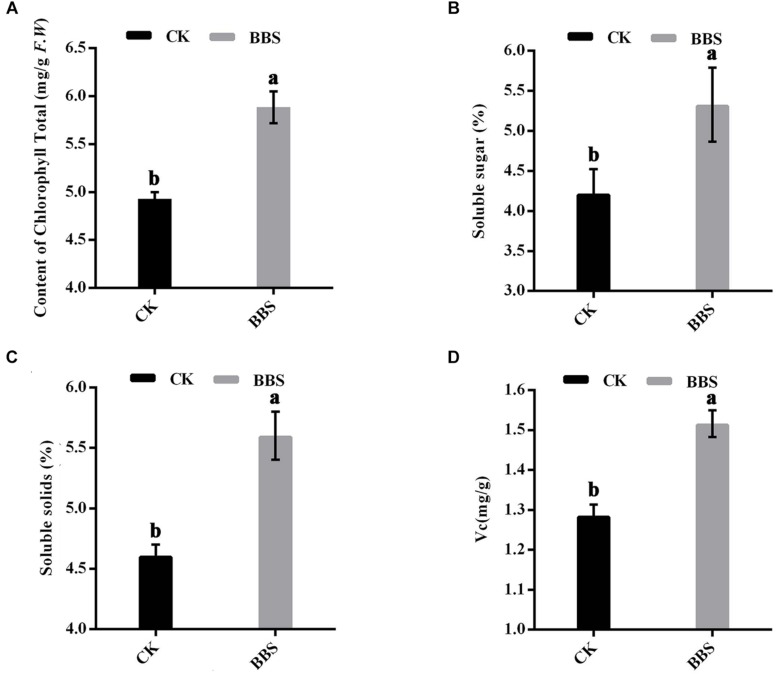
Effects of BBS management on content of chlorophyll **(A)** and fruit quality **(B–D)** of sweet pepper. Fruit quality of sweet pepper include content of soluble sugar **(B)**, soluble solids **(C)**, and Vitamin C **(D)**. Significant differences between different treatments are indicated as different letters on top of the data bars. The statistical analysis was determined by a Tukey’s Studentized Range (HSD) test: α = 0.05, *n* = 3.

It is generally known that photosynthesis determines the efficiency with which plants convert incoming sunlight to biomass; therefore, the chlorophyll content in the leaves was measured. Leaf total chlorophyll content was higher in BBS-treated plants than in control plants, at 5.88 mg/g in BBS-treated plants, versus 4.93 mg/g in the control (Figure 2A). Furthermore, PGPR agent has been reported to improve crop quality (Song et al., 2015). Thus, we determined the content of soluble sugar, soluble solid and vitamin C of mature sweet pepper at the harvest time. BBS-treated plants had significantly higher soluble sugar content (5.33%) than control plants (4.22%) (Figure 2B). Soluble solids content was higher in BBS-treated plants (5.60%) than in control plants (4.61%) (Figure 2C). BBS treatment increased the vitamin C content of sweet peppers relative to the control (Figure 2D). Therefore, BBS treatment improved the quality of sweet peppers.

### Effect of BBS on the Soil Properties

Previous studies demonstrated that application of PGPR improved the soil nutrient status (Gulnaz et al., 2017). BBS treatment affected the soil properties (Table 1). In the presence of BBS, soil TOM and TOC were significantly higher than in the control soil, at 123.81 g/kg (TOM) and 71.81 g/kg (TOC) in BBS-treated soil, versus 103.51 g/kg (TOM), and 60.04 g/kg (TOC) in the control. AN content was higher in BBS-treated soil (25.84 mg/kg) than in the control (19.02 mg/kg), whereas TN and NN contents did not differ between BBS-treated and control soil. TP and TK levels did not differ between the BBS-treated soil and control soil, whereas AP and AK levels were higher in BBS-treated soil (1.11 g/kg AP and 1.52 g/kg AK) compare than in control soil (0.82 g/kg AP and 1.38 g/kg AK). Therefore, BBS treatment improved soil properties.

**TABLE 1:** Physicochemical properties of soil samples under the different treatments.

	**TOM (g/kg)**	**TOC (g/kg)**	**TN (g/kg)**	**NN (mg/kg)**	**AN (mg/kg)**	**TP (g/kg)**	**AP (g/kg)**	**TK (g/kg)**	**AK (g/kg)**
CK	103.51 ± 1.66^b^	60.04 ± 0.96^b^	9.65 ± 0.83^a^	431.52 ± 2.01^a^	19.02 ± 0.12^b^	4.89 ± 0.18^a^	0.82 ± 0.01^b^	14.06 ± 0.16^a^	1.38 ± 0.01^b^
BBS	123.81 ± 3.83^a^	71.81 ± 2.22^a^	10.54 ± 0.76^a^	443.13 ± 5.57^a^	25.84 ± 1.01^a^	4.94 ± 0.18^a^	1.11 ± 0.04^a^	14.13 ± 0.13^a^	1.52 ± 0.02^a^

*Each treatment was replicated three times, data are presented as means of three replicates ± SD, and error bars represent SD for three replicates. Means with different letters have significant differences (*p* < 0.05; HSD test). TOM, total organic matter; TOC, total organic carbon; TN, total nitrogen; NN, nitrate nitrogen; AN, ammonia nitrogen; TP, total phosphorus; AP, available phosphorus; TK, total potassium; AK, available potassium.*

### Effect of Microbial Community Assemblages by BBS on Disease Control

A previous study has shown that microbe additives change microbial communities (Trabelsi and Mhamdi, 2013). Here, we characterized and identified the complex microbial community by simultaneous DNA amplicon sequencing targeting the 16S rRNA gene in bacteria. In total of 326 683 sequences of 16S rRNA were extracted from treated and control soil samples; the number of high-quality bacterial sequences varied among samples from 54,069 to 54,783 (Supplementary Table S1). Furthermore, 3,615 bacterial OTUs were obtained, with a limited number at the 97% similarity cut-off level. The Good’s coverage index revealed 99.00–99.09% of bacteria was obtained in all samples (Supplementary Table S1). The results showed that the probability of gene sequence detection in soil samples was high, and the sequencing results could represent the real situation of soil bacterial community in our experiment. The rarefaction and OTU Rank-Abundance curves of all six samples indicated that there was a smaller variation in the total number of OTUs, and the database of 16S rRNA gene sequences were very abundant which reflected the vast majority of microbial information (Supplementary Figure S1). The dominant phyla across all samples were Proteobacteria, Acidobacteria, Bacteroidates, Chloroflexi, Actinobacteria, Gemmatimonadetes, Planctomycetes, accounting for more than 89% of the bacterial sequences (Supplementary Figure S2a). Among these seven phyla, the relative abundance of Proteobacteria is the largest in the rhizosphere, which is composed of Alphaproteobacteria, Betaproteobacteria, Gammaproteobacteria and Deltaproteobacteria (Supplementary Figure S2b).

**FIGURE 3:**
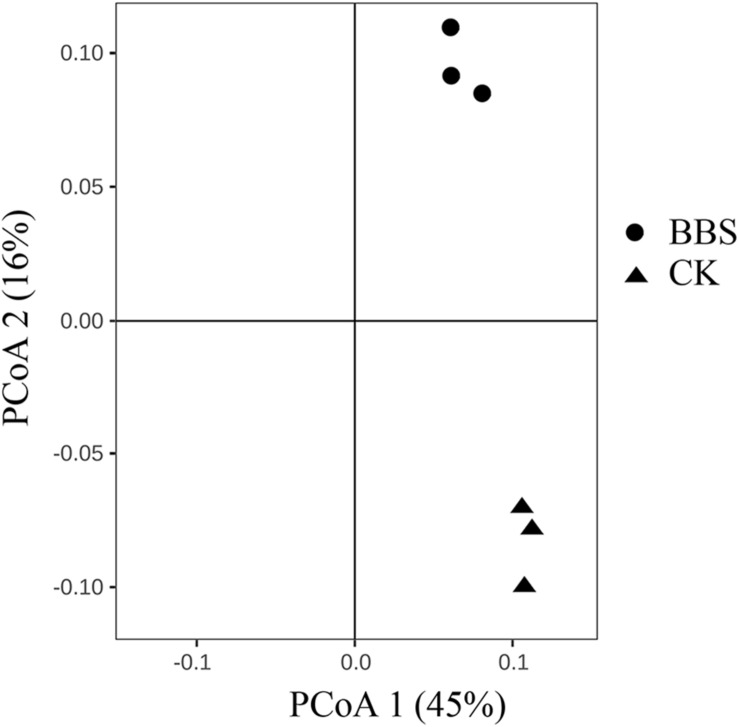
The bacterial microbial community compositions of the different treatments.

**FIGURE 4:**
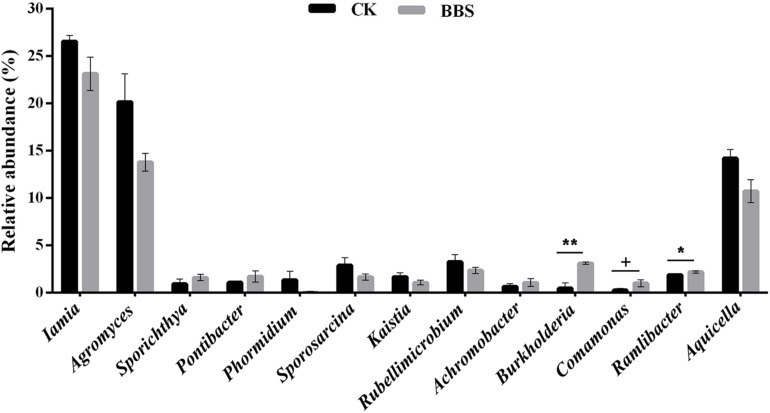
Relative abundance of disease-related genus in rhizosphere. Significant differences between different treatments are indicated as different letters on top of the data bars. The statistical analysis was determined by one-side *T*-test with 5% FDR (+*p* < 0.1, ^*^p < 0.05, and ^∗∗^*p* < 0.01).

Principal-coordinate analysis (PCoA) examination of between-sample variation (beta-diversity) based on Bray-Curtis distances revealed that rhizosphere bacterial communities, clustered by treatment, along the second component (Figure 3). Durán et al. (2018) demonstrated that managing microbial communities contributed to plant health. Spearman’s rank correlation coefficient revealed a clear positive correlation between phytophthora blight prevalence and the relative abundances of *Iamia* (*P* < 0.05), *Agromyces* (*P* < 0.01), *Kaistia* (*P* < 0.05), *Rubellimicrobium* (*P* < 0.05), *Sporosarcina* (*P* < 0.05), *Aquicella* (*P* < 0.05), and *Phormidium* (*P* < 0.05) (Table 2). In contrast, there were negative correlations between phytophthora blight prevalence and the relative abundances of *Sporichthya* (*P* < 0.05), *Achromobacter* (*P* < 0.05), *Burkholderia* (*P* < 0.01), *Comamonas* (*P* < 0.05), *Ramlibacter* (*P* < 0.05), and *Pontibacter* (*P* < 0.05) (Table 2). BBS treatment markedly increased the abundance of *Burkholderia* (moderated *t*-test, FDR, *p* < 0.01), *Comamonas* (moderated *t*-test, FDR, *p* < 0.1) and *Ramlibacter* (moderated *t*-test, FDR, *p* < 0.05), relative to the control (Figure 4 and Supplementary Figure S3). These results indicate that BBS modified the microbial community, contributing to the suppression of sweet pepper disease.

**TABLE 2:** Spearman’s rank correlation coefficient between rhizosphere abundant genus and disease incidence.

	**Disease incidence (%)**
*Iamia*	0.845^*^
*Agromyces*	0.897^∗∗^
*Sporichthya*	−0.847^*^
*Kaistia*	0.784^*^
*Rubellimicrobium*	0.787^*^
*Sporosarcina*	0.645^*^
*Achromobacter*	−0.739^*^
*Burkholderia*	−0.92^∗∗^
*Comamonas*	−0.864^*^
*Ramlibacter*	−0.854^*^
*Pontibacter*	−0.793^*^
*Aquicella*	0.930^*^
*Phormidium*	0.78^*^

Statistical significance at ^*^P < 0.05 and ^∗∗^*P* < 0.01.

### Effect of Microbial Community Assemblages on Soil Properties

**FIGURE 5:**
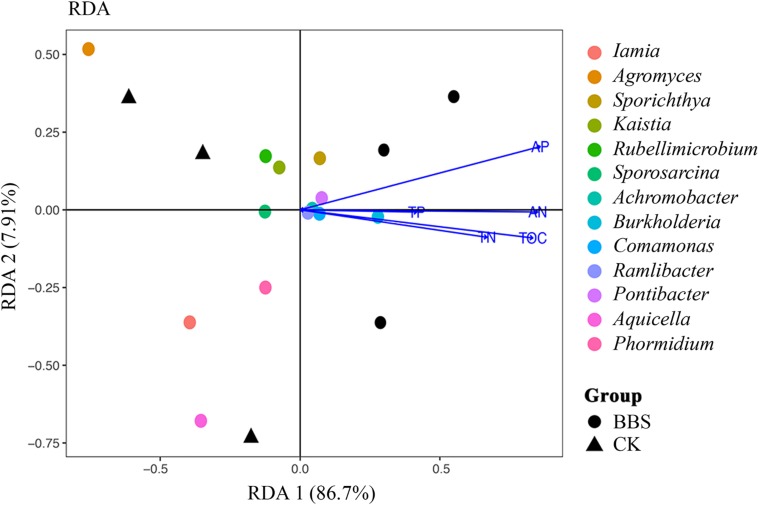
Redundancy analysis of soil properties, soil properties and analyzed rhizosphere soil genera bacterial. Soil property: TOC, total organic carbon; TN, total nitrogen; AN, ammonia nitrogen; TP, total phosphorus; AP, available phosphorus.

BBS treatment improved the soil chemical properties (Table 1). The Mantel test revealed striking relationships (*r* = 0.72, *P* < 0.05) between soil chemical properties and the abundances of the analyzed microbial genera. Examination of the relationship between the selected soil chemical properties and the abundances of the analyzed microbial genera (redundancy analysis) revealed that the two components explained the 91.41% variance, and BBS treatment was separated from the control treatment (Figure 5). BBS-treated soil samples were dominated by *Sporichthya*, *Achromobacter*, *Burkholderia*, *Comamonas*, *Ramlibacter*, and *Pontibacter*; bacterial abundance was related to TOC, TN, AN, TP, and AP (Figure 5). Therefore, BBS modified the microbial community thereby improving the soil properties. In summary, BBS shifted the microbe community to suppress soil-borne disease, increase sweet pepper crop yield and improve soil chemical properties.

## Discussion

Application of a consortium of three plant growth-promoting rhizobacteria (PGPR) strains (*Bacillus cereus* AR156, *B. subtilis* SM21, and *Serratia* sp. XY21) significantly reduced plant disease (Figure 1A); this is consistent with previous findings that PGPR strains can be used as biocontrol agents against plant diseases caused by soil-borne pathogens (Kloeppe et al., 1999; Haas and Défago, 2005). More importantly, BBS treatment improved sweet pepper fruit yield, raised its nutrient contents, and improved soil fertility (Figures 1B, 2A–D). These results confirm previous reports, in which PGPR was shown to act as both a biofertilizer and biocontrol agent (Mohapatra et al., 2015; Prasad et al., 2015).

Our finding that rhizosphere bacterial communities differed between the different treatments (Figure 3) is consistent with those of previous studies, which demonstrated that microbial amendments alter the rhizosphere microbiome (Saravanakumar et al., 2017). Based on Spearman’s rank correlation coefficient, the rhizosphere genera *Iamia*, *Agromyces*, *Kaistia*, *Rubellimicrobium*, *Sporosarcina*, *Aquicella*, and *Phormidium* were strongly and positively associated with sweet pepper disease. In contrast, negative associations between disease prevalence and the relative abundances of *Sporichthya*, *Achromobacter*, *Burkholderia*, *Comamonas*, *Ramlibacter*, and *Pontibacter* were observed (Table 2). Interestingly, BBS treatment significantly increased the abundance of *Burkholderia* (moderated *t*-test, FDR, *p* < 0.01), *Comamonas* (moderated *t*-test, FDR, *p* < 0.1), and *Ramlibacter* (moderated *t*-test, FDR, *p* < 0.05) (Figure 4), which were negatively associated with sweet pepper disease. Among the genera that occurred in our soil samples, *Burkholderia* has been used as a biological control agent against plant disease (Parke and Gurian-Sherman, 2001), *Comamonas* is reported to act against pathogenic fungi (El-Banna, 2007), and *Ramlibacter* appears to be important in adverse environments (Luca et al., 2011). Hence, we conclude that BBS shaped the rhizosphere microbial community by developing beneficial strains, thereby contributing to the suppression of sweet pepper disease.

BBS treatment improved soil chemical properties (Table 1), consistent with previous findings that PGPR can increase soil fertility (Sharma et al., 2017). The findings of our redundancy analysis (to evaluate the relationship between the selected soil chemical properties and the abundance of the analyzed microbial genera) suggest that BBS-treated soil samples were dominated by *Sporichthya*, *Achromobacter*, *Burkholderia*, *Comamonas*, *Ramlibacter*, and *Pontibacter*. Levels of TOC, TN, AN, TP, and AP were correlated with community composition (Figure 5). The abundance of the dominant genera were negatively associated with sweet pepper disease (Table 2), which suggest that BBS modified the microbial community to suppress soil-borne disease and improve soil chemical properties as well. As a result, BBS improved sweet pepper yield and improved the quality of fruit; this is consistent previous findings that high fertility soil promotes fruit yield and plant quality (Xu et al., 2001; Mahmoud et al., 2009). Liu et al. reported that cucumber roots could sense microbial signals releasing from additive PGPR *B. amyloliquefaciens* SQR9 and subsequently secrete tryptophan to recruit SQR9 which benefited the cucumber itself and prevented pathogen infection (Liu et al., 2017). Wang et al. demonstrated that plant root exudates are involved in *B. cereus* AR156 biocontrol ability against tomato bacterial wilt caused by *R. solanacearum*. Furthermore, plant root exudates have been reported to influence the soil bacterial community structure (Szoboszlay et al., 2016). Therefore, it will be interesting to understand the mechanism of the bacteria shaping rhizosphere bacterial communities by regulating plant root exudates.

## Data Availability

The datasets generated for this study can be found in the Sequence Read Archive (SRA) NCBI database 162 (accession number PRJNA526286).

## Author Contributions

L-NZ and J-HG designed the experiments. L-NZ performed the experiments with assistance from QH, X-QD, Y-SX, QL, and H-ML. D-CW, L-NZ, and J-HG wrote the manuscript.

## Conflict of Interest Statement

QL and H-ML were employed by the Wuhan Kernel Bio-tech Co., Ltd. The remaining authors declare that the research was conducted in the absence of any commercial or financial relationships that could be construed as a potential conflict of interest.
