# Supplementary material for: Consortium of Plant Growth-Promoting Rhizobacteria Strains Suppresses Sweet Pepper Disease by Altering the Rhizosphere Microbiota
Source: Front Microbiol. 2019 Jul 23;10:1668. doi: 10.3389/fmicb.2019.01668 (PMC6664061; doi:10.3389/fmicb.2019.01668)
Supplement: Supplementary file 1 [file Table_1.docx]

TABLE S1 | Retained sequences, OUT number and alpha diversity statistics that were used for further analysis after removing short, ambiguous, and low-quality reads of soil samples in this study.

| **Sample Name** | **Retained sequences** | **OUT number** | **Sobs** | **Chao 1** | **ACE** | **Shannon's diversity** | **Simpson's diversity** | **Good’s coverage** |
| --- | --- | --- | --- | --- | --- | --- | --- | --- |
| CK1 | 54069 | 2754 | 2754 | 3158.63 | 3149.31 | 6.7045 | 0.002814 | 0.989994 |
| CK2 | 54235 | 2791 | 2791 | 3147.64 | 3117.07 | 6.7314 | 0.002824 | 0.990818 |
| CK3 | 54784 | 2785 | 2785 | 3250.92 | 3168.48 | 6.7229 | 0.002863 | 0.99007 |
| BBS1 | 54603 | 2791 | 2791 | 3172.80 | 3189.99 | 6.6996 | 0.002873 | 0.990074 |
| BBS2 | 54455 | 2766 | 2766 | 3092.12 | 3109.10 | 6.7087 | 0.002825 | 0.990836 |
| BBS3 | 54537 | 2821 | 2821 | 3137.29 | 3150.65 | 6.7439 | 0.002863 | 0.990942 |


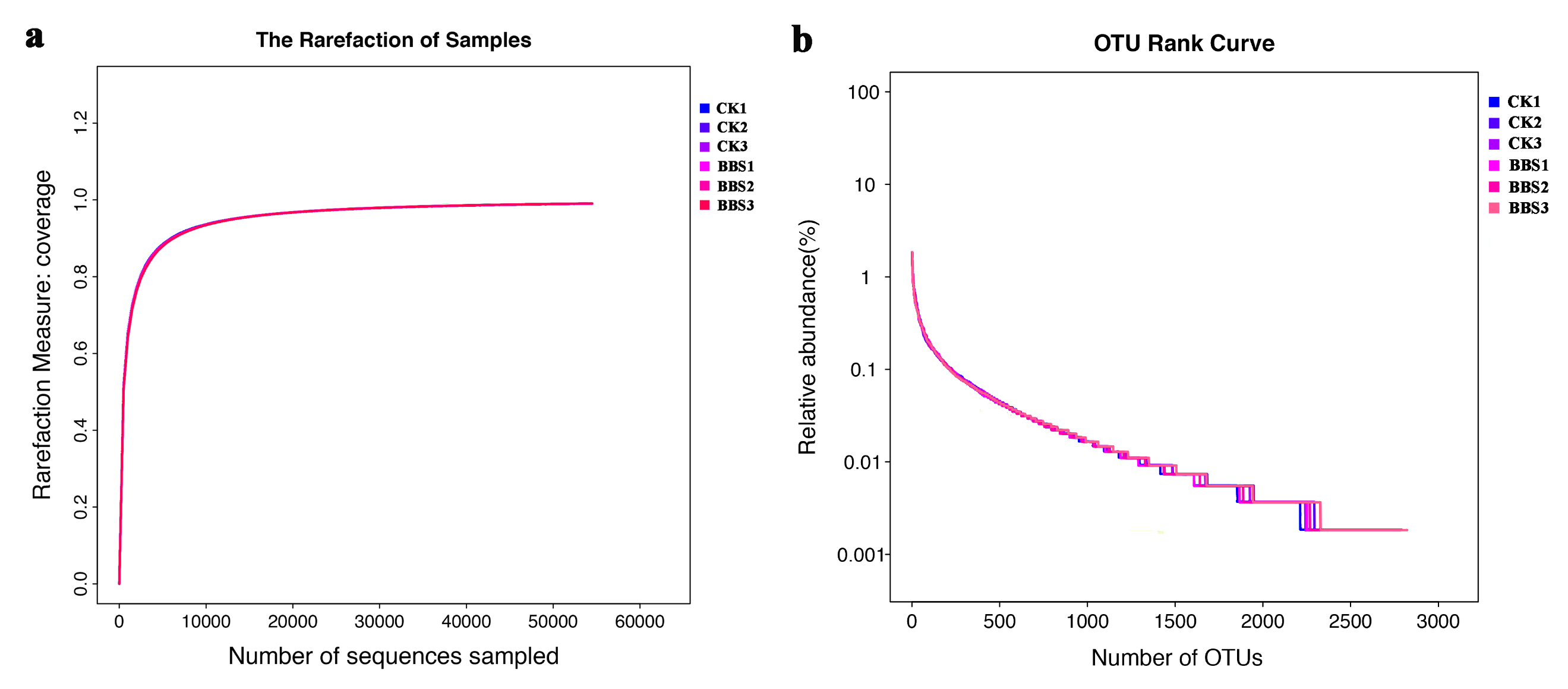


FIGURE S1 | The curves of the OTUs obtained from six samples. Rarefaction curves (a), OTU rank-abundance curves (b).


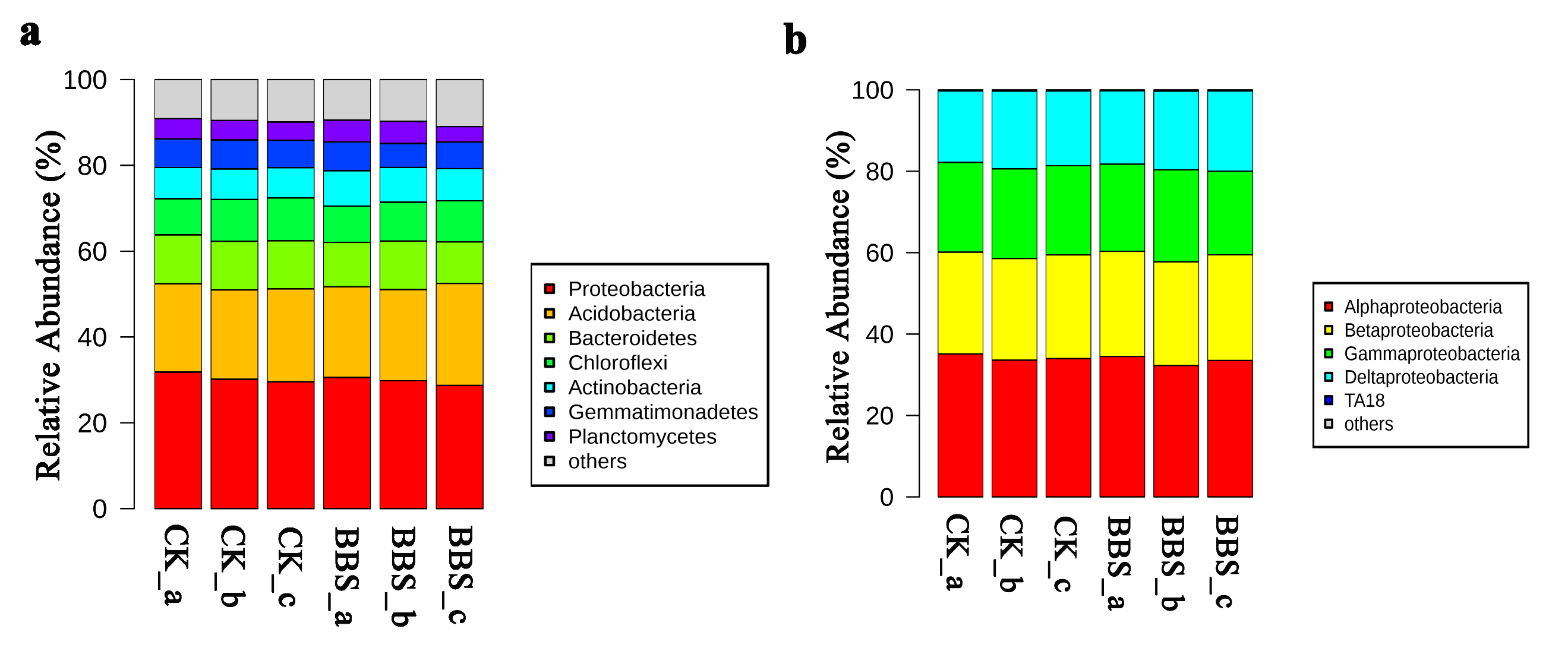


FIGURE S2 | Taxonomic composition of six samples. The composition was classified at phylum (a), The composition of Proteobacteria was classified at class (b).


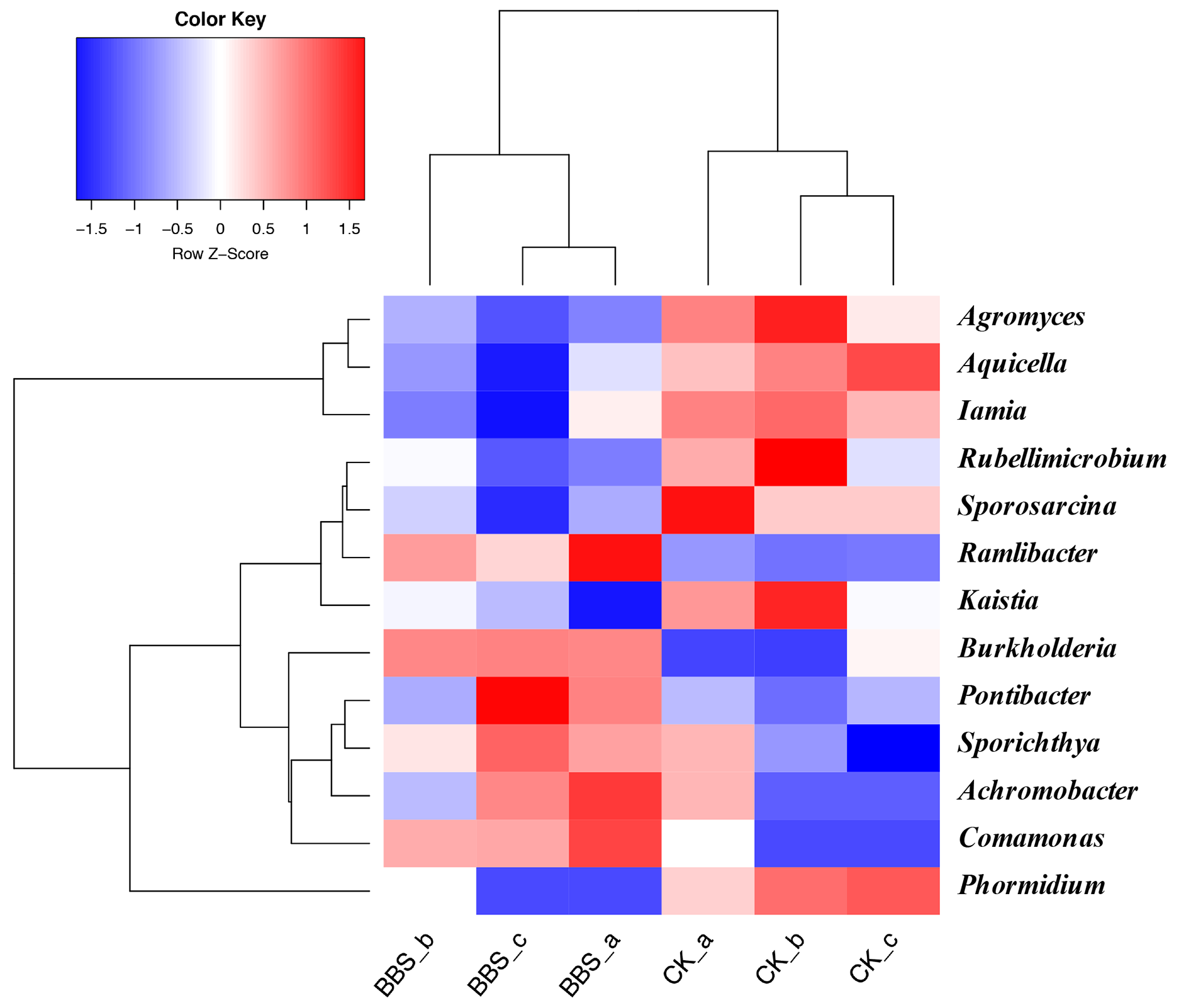


FIGURE S3 | Heatmap based on relative abundance of selected rhizosphere soil microbial genera and different soil samples. Vertical columns represent samples; horizontal rows represent selected rhizosphere soil microbial genera.
